# Onset of vitiligo in a patient with acquired secondary hypogonadism under treatment with testosterone gel 2%: inside the pathogenesis^☆^^☆☆^

**DOI:** 10.1016/j.abd.2020.02.010

**Published:** 2020-07-15

**Authors:** Giovanni Paolino, Pietro Bearzi, Santo Raffaele Mercuri

**Affiliations:** Department of Dermatology, IRCCS Ospedale San Raffaele, Milan, Italy

Dear Editor,

A 61-year-old man presented with a three-month history of symmetric depigmented lesions on the hands and perilabial region (Figure 1, Figure 2). No other anatomical area was involved. Under Wood’s light the lesions appeared bright white and sharply demarcated, confirming the diagnosis of vitiligo. His personal and family medical history was negative for autoimmune disorders. The patient reported that eight months before, a diagnosis of acquired secondary idiopathic hypogonadism due to aging was made (low levels of testosterone; normal LH and FSH levels; negative MRI of the hypothalamic-pituitary region and normal body mass index). Laboratory investigations and analysis of autoimmunity showed no alterations. Thyroid function was normal and thyroid antibodies were absent. According to this diagnosis, he started treatment with topical testosterone 2% gel once a day for six months, which he applied at level of the chest and thighs. He noticed the first depigmentation area on the hands, during the fourth month of treatment. For vitiligo areas, pimecrolimus 1% cream was prescribed. After six months of follow-up, the treated vitiligo areas are stable and no further depigmented areas arose. The patient continues to perform the endocrinological consultations with good hormonal compensation.

**Figure 1:**
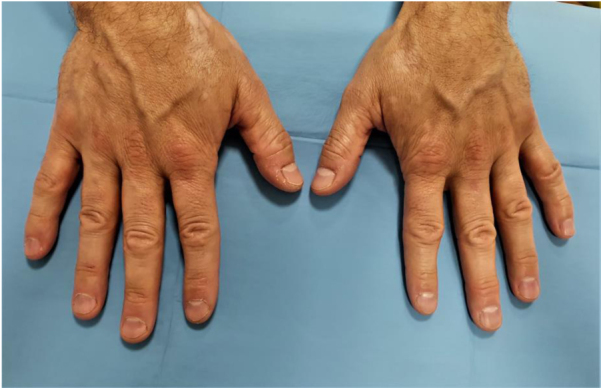
Symmetric macules of vitiligo on the dorsomedial part of the hands.

**Figure 2:**
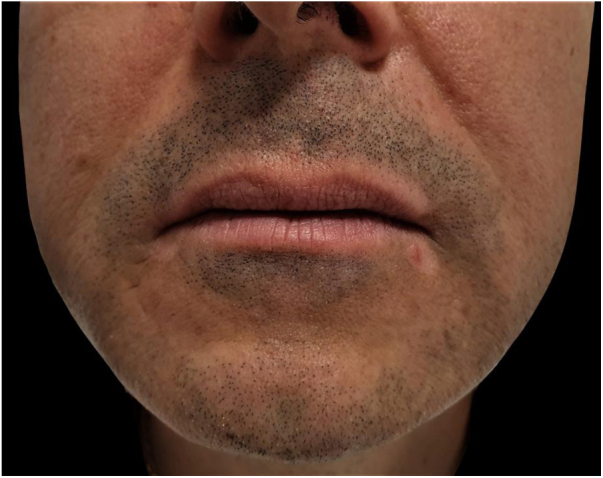
Perilabial depigmentation.

The etiology of vitiligo is still unclear.^1^ Several hypotheses have been proposed to explain the causes of melanocyte dysfunction.^1^ In this regard, Kotb et al. also investigated the hormonal theory.^1^ Specifically, they found a significant decrease in testosterone levels in the serum of males in both active and stable vitiligo groups when compared to controls, and in the active vitiligo group compared to the stable vitiligo group; besides, they did not find significant difference in testosterone levels between males and females in the active vitiligo group.^1^ Furthermore, testosterone can be also involved in the downregulation of the inflammation. Indeed, testosterone suppresses immunity, affecting T-cell immunity by inhibiting T-helper 1 differentiation, justifying the sex differences in the response to viruses and autoimmunity.^2^ All these findings confirm that hypogonadism may explain the predisposition for the onset of vitiligo in the present patient.

Drug-induced vitiligo is a rare side effect of several systemic and topical drugs.^3^ In these cases, skin depigmentation is indistinguishable from vitiligo and appears to be due to activation of melanocyte-specific autoimmunity; for these reasons, this phenomenon should be known as drug-induced vitiligo, rather than with the less accurate term “drug-induced depigmentation.”^4^ Goldstein et al. reported that testosterone may interact with tyrosine hydroxylase activity; besides, it is known that agents that interact with tyrosinase activity can paradoxically disrupt melanin production, by inducing the cellular stress response with inflammation and autoimmune destruction of melanocytes.^5^ However, to date, there is no evidence of testosterone-induced vitiligo.

As far as the authors know, the present case represents the first clinical confirmation of the onset of vitiligo in a patient with hypogonadism, supporting the thesis that hypogonadism can be associated with vitiligo. At the same time, there is insufficient evidence to support a role of testosterone replacement therapy in the development of vitiligo. Indeed, no areas of depigmentation were observed at the testosterone application sites (chest and thighs). Further studies are needed to investigate the role of testosterone in pigmentation, also extending the research to possible new therapeutic implications.

## Financial support

None declared.

## Authors' contributions

Giovanni Paolino: Drafting and editing of the manuscript; intellectual participation in the propaedeutic and/or therapeutic conduct of the studied cases; critical review of the literature; critical review of the manuscript.

Pietro Bearzi: Drafting and editing of the manuscript; intellectual participation in the propaedeutic and/or therapeutic conduct of the studied cases; critical review of the literature; critical review of the manuscript.

Santo Raffaele Mercuri: Approval of the final version of the manuscript; intellectual participation in the propaedeutic and/or therapeutic conduct of the studied cases; critical review of the literature; critical review of the manuscript.

## Conflicts of interest

None declared.

## References

[1] Kotb El-Sayed M.I., Abd El-Ghany A.A., Mohamed R.R. (2018). Neural and endocrinal pathobiochemistry of vitiligo: comparative study for a hypothesized mechanism. Front Endocrinol (Lausanne)..

[2] Kissick H.T., Sanda M.G., Dunn L.K., Pellegrini K.L., On S.T., Noel J.K. (2014). Androgens alter T-cell immunity by inhibiting T-helper 1 differentiation. Proc Natl Acad Sci U S A..

[3] Curzytek K., Pietowska J., Spiewak R. (2007). Drug-induced vitiligo: a metanalysis of reported cases. J Physiol Pharmacol..

[4] Harris J.E. (2017). Chemical-induced vitiligo. Dermatol Clin..

[5] Goldstein M.E., Tank A.W., Fossom L.H., Hamil R.W. (1992). Molecular aspects of the regulation of tyrosine hydroxylase by testosterone. Brain Res Mol Brain Res..

